# The Qixiangzhan eruption, Changbaishan-Tianchi volcano, China/DPRK: new age constraints and their implications

**DOI:** 10.1038/s41598-022-27038-5

**Published:** 2022-12-28

**Authors:** Bo Pan, Shanaka L. de Silva, Martin Danišík, Axel K. Schmitt, Daniel P. Miggins

**Affiliations:** 1 Jilin Changbaishan Volcano National Observation and Research Station, Institute of Geology, China Earthquake Administration, Beijing, 100029 China; 2 College of Earth, Ocean, and Atmospheric Sciences, Oregon State University, Corvallis, OR 97331 USA; 3 John de Laeter Centre, Curtin University, Perth, WA 6845 Australia; 4 Institut für Geowissenschaften, Universität Heidelberg, Heidelberg, 69120 Germany

**Keywords:** Natural hazards, Geology, Palaeomagnetism, Volcanology

## Abstract

Zircon double dating (ZDD) of comendite lava reveals an eruption age of 7.0 ± 0.9 ka for the Qixiangzhan eruption (QXZ), Changbaishan-Tianchi volcano, China/DPRK. This age is supported by new ^40^Ar/^39^Ar sanidine experiments and a previous age control from charcoal at the base of the QXZ. The revised age supports correlations with distal ash in Eastern China and Central Japan and establishes a significant (estimated at Volcanic Explosivity Index 5+) eruption that may provide a useful Holocene stratigraphic marker in East Asia. The new age indicates that the QXZ lava does not record a ca. 17 ka Hilina Pali/Tianchi geomagnetic field excursion but rather a heretofore unrecognized younger Holocene excursion at ca. 7–8 ka. Comparison between U–Th zircon crystallization and ZDD as well as ^40^Ar/^39^Ar sanidine ages indicates a protracted period of accumulation of the QXZ magma that extends from ca. 18 ka to the eruption age. This connotes an eruption that mixed remobilized early formed crystals (antecrysts) from prior stages of magma accumulation with crystals formed near the time of eruption. Based on these results, a recurrence rate of ca. 7–8 ka for the Changbaishan-Tianchi magma system is found over the last two major eruption cycles.

## Introduction

**Figure 1:**
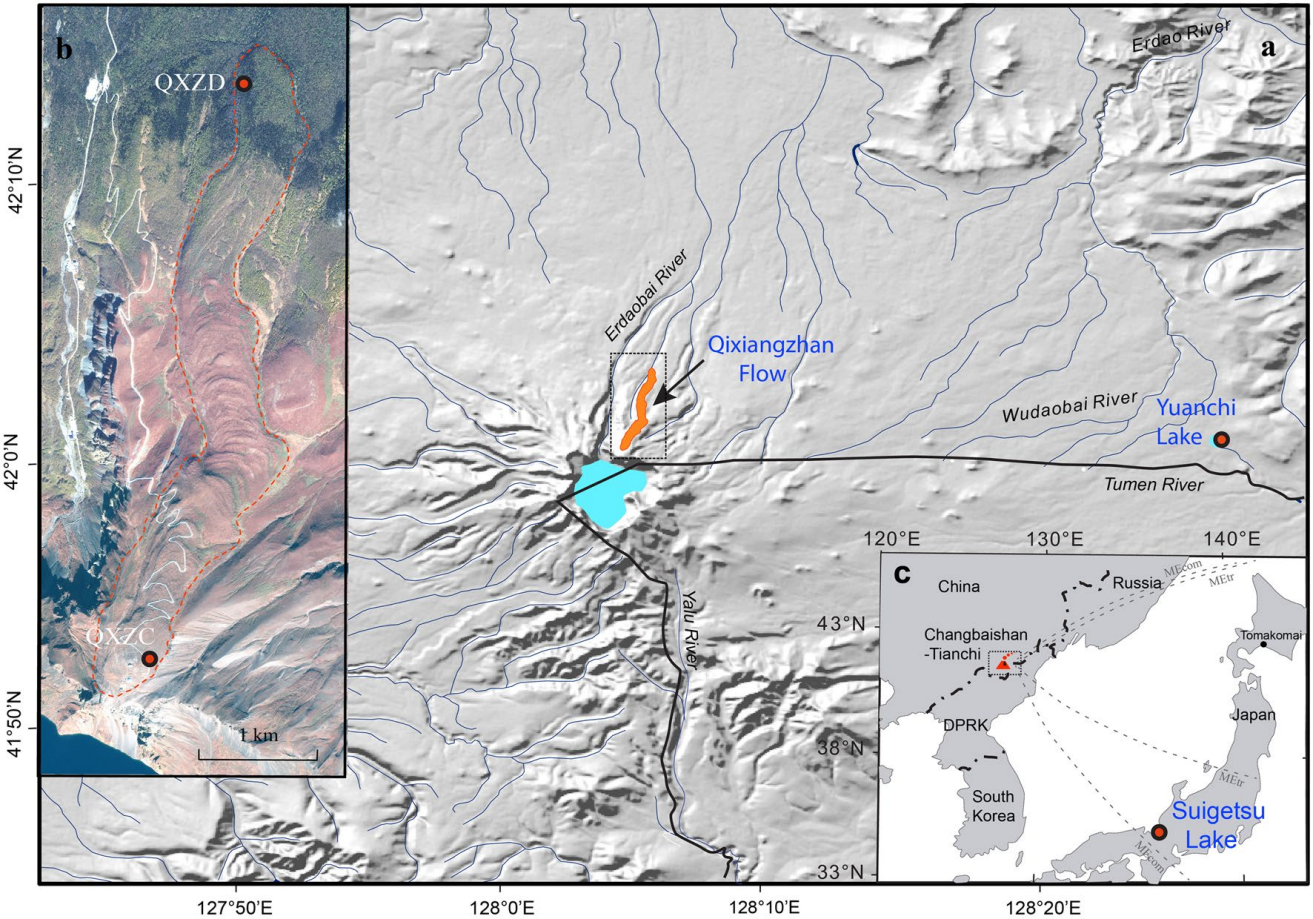
Geographic context and details of the Qixiangzhan (QXZ) eruption from Changbaishan-Tianchi volcano. (**a**) Shaded relief of the Changbaishan-Tianchi volcano and local environs showing the location and distribution of the Qixiangzhan lava flow on the north slope of Changbaishan-Tianchi caldera. Solid black line is the international border between China and the DPRK. (**b**) Satellite image of the Qixiangzhan lava flow and showing our sample locations. (**c**) Broader regional context of Changbaishan-Tianchi volcano showing locations where correlatives of the tephra/ash of the Qixiangzhan eruption have been located.

Accurate ages of Quaternary eruptions are crucial for reliable delineation of volcanic histories and associated time scales, with implications for geodynamics and volcanic hazards, as well as the development and evolution of magmatic systems. Widespread tephra deposits are also important chronostratigraphic markers and thus accurate eruption ages are crucial for constraining Quaternary stratigraphy. However, it is increasingly becoming clear that obtaining robust and precise direct ages (recorded in juvenile material) for eruptions < 100 ka can be very challenging compromising accurate volcanic chronology in this critical time period. The Qixiangzhan eruption (QXZ) of the Changbaishan-Tianchi volcano (CBS-TC) on the China/Democratic People's Republic of Korea (DPRK) border (Fig. 1) exemplifies many of these challenges as different geochronological methods have resulted in often conflicting eruption age interpretations^1–3^. The QXZ event is a key eruption in NE China/Japan as it apparently records a world-wide geomagnetic field excursion within its lava and welded pyroclastic deposits. This excursion has been variously correlated to the ca. 120-kyr Blake event^4^, and more recently, to the Hilina Pali excursion resulting in a postulated new “Hilina Pali/Tianchi” paleomagnetic event^1^. Adequate eruption age assignment has gained even more significance due to the recent correlation of the QXZ with distal ash deposits in China (Lake Yuanchi—30 km away^2^) and Japan (Lake Suigetsu—900 km away^5^), connoting an explosive eruption of regional significance with the potential to help correlate the northern hemispheric “8.2 ka event”, an abrupt cooling transition at ca. 8.2 kyr BP (ref.^6^). The validation of any of these potential associations depends on the precise timing of the QXZ.

To date, direct dating of juvenile materials from the QXZ has returned variable results and the most recently promoted age of ca. 8.1 ka is based on indirect dates from radiocarbon-14 (^14^C), stratigraphy and tephrochronology^2,5^. In this contribution, we address this challenge by using the combined application of (U–Th)/He and ^238^U/^230^Th disequilibrium dating of zircon^7^, known as zircon double dating (ZDD)^8^, indicating a new direct age of 7.0 ± 0.9 ka for the QXZ. In addition, we have conducted high precision ^40^Ar/^39^Ar sanidine single crystal incremental heating experiments that yield ages ranging from 7.2 ± 1.3 to 14.3 ± 0.7 ka that support this new young age. The outcome of this study has important implications for regional tephrochronology, geomagnetic field history, deglaciation as an eruption trigger, and the magmatic history of the Changbaishan-Tianchi volcano, and dating of Quaternary volcanic eruptions.

## The Qixiangzhan eruption, Changbaishan-Tianchi volcano, China/DPRK

Changbaishan-Tianchi volcano (or Baegdusan, Paektusan in the DPRK; Baitoushan in Japanese; Fig. 1) is the source of one of the most powerful explosive eruptions (VEI 6 to 7) in the Holocene, the Millennium Eruption (ME) of 946–947 CE (Common Era)^3,9,10^. The eruption that preceded the ME is the Qixiangzhan eruption^3^. In addition, the QXZ has been demonstrated to overlie the much older Tianwenfeng “Yellow Pumice” eruption which has been geochemically correlated with the 50.6 ka B-J tephra layer in the Japan Sea^3^. Most descriptions of the proximal QXZ focus on the massive lava flow that extends ~ 5.4 km northwards downslope from the CBS-TC summit that Pan et al.^11^ described as clastogenic, implying explosive fountain fed activity. However, Pan et al.^12^ also documented interbedded pyroclastic horizons throughout the lava, many of which are welded throughout the proximal QXZ stratigraphy (Fig. S1). These confirm significant explosive activity accompanied the local effusion of lava.

The confusion with the age of the QXZ is demonstrated in a summary of 16 previously published ages for the QXZ ranging from 87 to 4 ka^1–3,5,13–20^ (Table 1). Note that there and elsewhere in this paper, unless otherwise stated, uncertainties are stated at the 2σ level. Ignoring the 1980s era K–Ar experiments that are clearly biased by a lack of correction for unsupported ^40^Ar^13^, the rest of the ages are all younger than 20 ka. All the direct dating experiments are of material from the proximal lava flow and consist of a series of modern ^40^Ar/^39^Ar experiments on sanidine that yield ages ranging from 19.7 ± 2.8 to 7.6 ± 0.2 ka^14,17^, Uranium-series disequilibrium experiments yielding ages ranging from 18.4 ± 1.4 to 4.3 ± 0.4 ka^15^, a U–Th disequilibrium zircon crystallization age of 12.2 ± 1.7 ka^16^, and Electron Spin Resonance method (ESR) and Thermoluminescences (TL) age determinations that have yielded ages of 3.9 ± 0.5 ka^19^ and 3.5 ± 0.3 ka^20^ respectively (all sources are given in Table 1).

**Table 1:** Previous geochronological data for the Qixiangzhan eruption from the Changabaishan-Tianchi volcano.

No	Sample #	Material	Methods	Ages	Location
1	TK13	Sanidine	K–Ar	87.6 ± 15ka^13^	Proximal Lava flow
2	CT6-1	Sanidine	K–Ar	80.4 ± 4.1ka^13^	Proximal Lava flow
3	10CB-7	Sanidine	^40^Ar/^39^Ar	19.7 ± 2.8 ka^14^	Proximal Lava flow
4	C-5	Whole rock	U-TIMS	18.4 ± 1.4 ka^15^	Proximal Lava flow
5	CB-01	Sanidine	^40^Ar/^39^Ar	17.1 ± 0.9 ka^1^	Proximal Lava flow
6	QXZ	Zircon	U–Th	12.2 ± 1.1 ka^16^	Proximal Lava flow
7	10CB-4	Sanidine	^40^Ar/^39^Ar	11.1 ± 0.4 ka^14^	Proximal Lava flow
8	QIZ	Sanidine	^40^Ar/^39^Ar	7.6 ± 0.2 ka to 10.5 ± 0.5 ka^17^	Proximal Lava flow
9	C-8	Whole rock	U-TIMS	9.7 ± 1.5 ka^15^	Proximal Lava flow
10	YC -162	Tephra layer	TC, C14	~ 8.1 ka^2^	Yuanchi lake, China
11	TQC1	Burnt branch	C14	7.37 ± 0.03 ka^3^	Proximal Lava flow
12	B-Sg-08	Tephra layer	TC, C14	8.166–8.099 ka^5^	Sugietsu lake, Japan
13	C-6	Whole rock	U-TIMS	4.3 ± 0.4 ka^15^	Proximal Lava flow
14	97P6	Stratigraphy	SS	< 4 ka^18^	Proximal Lava flow
15	C-5	Quartz	ESR	3.93 ± 0.5 ka^19^	Proximal Lava flow
16	Y-5	Sanidine	TL	3.53 ± 0.30 ka^20^	Proximal Lava flow

Ordered by age from old to young. C14-Radiocarbon dating; TL-Thermoluminescence; ESR-Electron Spin Resonance; U-TIMS-U series TIMS; TC-Tephrochronology; SS-Stratigraphy sequence.

Recently, several works focused on tephrochronology have demonstrated geochemical correlation of the Qixiangzhan comendite lava glass composition with the YC-162 tephra from Lake Yuanchi^2^, ~ 30 km to the east of the CBS-TC, and the SG14-1058 (sample B-Sg-08) tephra recorded in Lake Suigetsu, Japan, ~ 900 km to the SE of the CBS-TC^5^. The SG14-1058 tephra containing layers from Lake Suigetsu and the YC-162 tephra have yielded ^14^C ages of 8166–8099 and 8831–8100 cal yr BP (95% confidence), respectively^2,5^. A younger ^14^C age of 7370 ± 30 cal yr BP (95% confidence) from burnt vegetation (charcoal) adhered to the base of the QXZ lava was reported by Pan et al.^3^ on the basis of which they proposed a < 8 ka age for the QXZ.

## Results and interpretation

**Figure 2:**
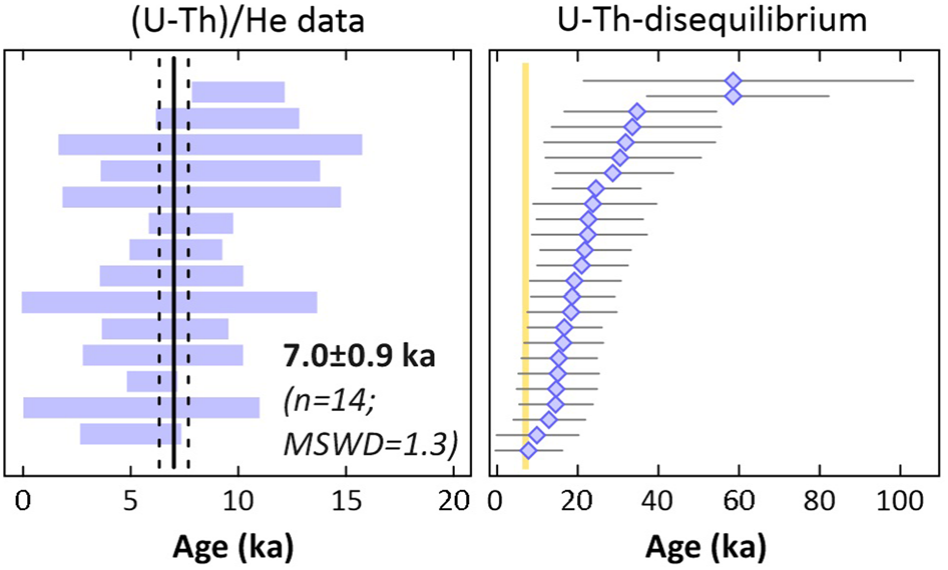
Graphical summary of zircon double dating results: Left panel—ranked order plot of alpha ejection and disequilibrium corrected zircon (U–Th)/He dates displayed as 2σ error bars. Weighted mean (solid black line) and 95% confidence interval (dashed black lines) represent our best ZDD estimate for the time of eruption and its uncertainty (or ZDD eruption age). Right panel—ranked order plot of zircon U–Th ages with 1σ analytical uncertainties. Deep yellow bar indicates the 95% confidence interval of the ZDD eruption age. Note that U–Th ages provide maximum eruption age and the youngest U–Th ages overlap within uncertainty with the ZDD eruption age, providing additional confidence in the data.

25 zircon crystals from sample QXZD (Fig. 1) have yielded U–Th disequilibrium model ages ranging from ca. 7.7 to 59 ka (Fig. 2; Table S1). 18 of these zircons were analyzed for (U–Th)/He; 4 crystals yielded anomalously old (U–Th)/He dates which are disregarded for age calculation as they are clearly outliers; 3 of the anomalously old (U–Th)/He dates (i.e. crystals QXZD-5, -14, -21) are older than their corresponding crystallization ages and therefore are interpreted as analytical outliers likely resulting from grain imperfection such as undetected mineral or fluid inclusions. Crystal QXZD-25 with (U–Th)/He date of 53 ± 4.0 ka and crystallization age 58.5 ± 11.2 ka (i.e. the last one from the group of anomalously old (U–Th)/He dates) is a statistical outlier based on a modified 2-sigma criterion^21^ and likely represents an inherited zircon from an older magmatic cycle, maybe the underlying Tianwenfeng “Yellow Pumice” eruption.

The remaining 14 zircons form a uniform (U–Th)/He age population with a weighted average of 7.0 ± 0.9 ka (Fig. 2; Table 2).

**Table 2:** Summary of the Zircon double dating data results for the Qixiangzhan eruption.

Sample code/ crystal number	^232^Th (ng)	± (%)	^238^U (ng)	± (%)	^147^Sm (ng)	± (%)	^4^He (ncc)	± (%)	TAU (%)	Th/U	Raw He age (ka)	± 1σ (ka)	Ft	± (%)	Ft.-cor. He age (ka)	± 1σ (ka)	U–Th (ka)	± 1σ (ka)	D_230_	Diseq.-cor. He age (ka BP)	± 1σ (ka)
**QXZD**																					
QXZD-24	3.464	1.9	4.097	2.3	0.02484	1.8	0.0029	14.4	14.5	0.84	4.9	0.7	0.84	5	5.8	0.9	21.6	5.2	0.5150	7.1	1.1
QXZD-20	2.126	1.4	2.256	1.8	0.02149	2.9	0.0016	22.1	22.2	0.94	4.8	1.1	0.82	5	5.8	1.3	34.7	9.4	0.5739	6.6	1.5
QXZD-23	1.825	1.4	2.183	1.8	0.01945	2.6	0.0014	27.2	27.3	0.83	4.3	1.2	0.81	5	5.3	1.5	15	4.6	0.5091	6.5	1.9
QXZD-9	1.345	1.9	1.531	2.3	0.01133	5.2	0.0012	38.6	38.6	0.87	5.2	2.0	0.76	5	6.9	2.7	18.2	5.5	0.5353	8.3	3.3
QXZD-2	1.521	1.4	1.768	1.8	0.01233	4.6	0.0014	29.0	29.1	0.85	5.5	1.6	0.77	5	7.2	2.1	19.1	5.3	0.5240	8.7	2.6
QXZD-8	1.387	1.4	1.709	1.8	0.00876	5.7	0.0013	39.9	39.9	0.81	5.4	2.1	0.77	5	6.9	2.8	14.6	3.2	0.4941	8.7	3.6
QXZD-25*	1.421	1.9	1.755	2.3	0.01780	3.7	0.0108	5.2	5.6	0.80	42.4	2.4	0.80	5	53.0	4.0	58.5	11.2	0.4932		
QXZD-22	2.541	1.4	2.975	1.8	0.03296	1.5	0.0027	16.1	16.1	0.85	6.2	1.0	0.83	5	7.4	1.3	12.7	4.0	0.5201	9.5	1.7
QXZD-5*	1.041	1.4	1.241	1.8	0.00750	4.7	0.0238	2.8	3.2	0.83	131.7	4.2	0.80	5	163.7	9.7	22.4	6.7	0.5109		
QXZD-1	0.687	1.4	0.845	1.8	0.01641	5.9	0.0005	48.5	48.5	0.81	4.2	2.0	0.76	5	5.5	2.7	15.2	4.2	0.4950	6.8	3.5
QXZD-4	5.492	1.4	6.030	1.8	0.10051	1.1	0.0034	7.6	7.7	0.90	3.8	0.3	0.75	5	5.0	0.5	18.5	4.7	0.5547	6.0	0.6
QXZD-6	2.014	2.0	1.255	2.3	0.01919	4.9	0.0008	23.0	23.0	1.59	3.9	0.9	0.78	5	5.1	1.2	16.3	4.4	0.9779	5.0	1.2
QXZD-11	0.854	1.4	0.923	1.8	0.01962	9.4	0.0004	55.4	55.5	0.92	3.1	1.7	0.71	5	4.3	2.4	33.5	10.5	0.5637	5.5	2.8
QXZD-13	2.430	1.4	2.052	1.8	0.10058	2.8	0.0018	11.8	11.9	1.18	5.6	0.7	0.77	5	7.3	0.9	30.4	9.7	0.7213	7.8	1.0
QXZD-21*	2.010	1.4	2.008	1.8	0.06966	1.5	0.0221	2.1	2.6	0.99	73.2	1.9	0.81	5	90.2	5.1	16.5	4.1	0.6098		
QXZD-18	2.198	2.0	3.425	2.3	0.05183	1.5	0.0028	9.4	9.6	0.64	5.9	0.6	0.81	5	7.3	0.8	22.6	6.3	0.3909	10.0	1.1
QXZD-19	1.397	1.4	1.637	1.8	0.04745	2.2	0.0011	24.1	24.2	0.85	4.6	1.1	0.80	5	5.7	1.4	20.9	5.1	0.5200	6.9	1.7
QXZD-14*	1.910	1.4	1.856	1.8	0.07953	1.9	0.0070	4.2	4.5	1.02	25.0	1.1	0.80	5	31.4	2.1	24.4	4.9	0.6269		
							**Weighted mean (in ka BP) ± 95% conf. (in ka) (MSWD):**								**7.01 ± 0.87 (1.3)**						
**FCT (Fish Canyon Tuff) zircon**																					
ZFC-1	0.705	2.2	1.437	2.5	0.00080	8.8	4.7726	0.9	2.4	0.49	24.4	0.6	0.86	5	28.4	1.6					
ZFC-2	1.179	1.5	1.380	2.0	0.00136	16.8	4.5870	0.9	1.9	0.85	22.7	0.4	0.79	5	28.8	1.5					
ZFC-4	1.070	1.5	1.349	2.0	0.00050	7.1	4.5601	0.9	1.9	0.79	23.4	0.4	0.81	5	28.9	1.5					
ZFC-6	0.940	2.4	1.421	2.7	0.00054	22.3	4.3674	0.9	2.5	0.66	21.8	0.5	0.78	5	28.0	1.6					
							**Weighted mean (in ka BP) ± 95% conf. (in ka) (MSWD):**								**28.5 ± 1.5 (0.06)**						

TAU—total analytical uncertainty; F_t_—alpha ejection correction factor calculated after Farley et al.^49^ for homogeneous distribution of parent nuclides; uncertainty on Ft factors was arbitrarily set to 5% and 10% for crystals with Ft ≥ 0.6 and Ft < 0.6, respectively, after Ehlers and Farley (2003) and propagated in quadrature into final age uncertainty; D_230_—Th zircon-melt fractionation factor calculated after^50^ using the Th/U ratios of analyzed bulk zircon crystals and whole rock Th/U (1.63); Diseq.-cor. (U–Th)/He age (ka)—disequilibrium corrected (U–Th)/He age calculated by MCHeCalc program^53^ assuming D_231_ = 3.3 (i.e., an average of ƒ_Pa/U_ values published by^54–56^; Eruption ages were calculated as error weighted average by Isoplot 4.15 Excel add-in^21^. Crystals marked with asterisk were disregarded as statistical outliers because of their anomalously old (U–Th)/He date that is likely related to inclusions or other imperfection of the crystals.

The ZDD eruption age we have obtained (7.0 ± 0.9 ka) is significantly younger than the ^40^Ar/^39^Ar in sanidine ages of Yang et al.^14^ and Singer et al.^1^ but is concordant with the youngest sanidine age of 7.6 ± 0.2 ka reported by Heizler et al.^17^ from the same sample QXZC. To verify this, six large (> 850 μm) sanidine crystals from sample QXZC were chosen for analysis by the single crystal incremental heating (SCIH) ^40^Ar/^39^Ar technique. These yield individual crystal plateau ages ranging from 14.3 ± 0.7 to 7.2 ± 1.3 ka. The plateau ages are concordant with their respective normal, inverse, and total fusion ages (Fig. S2; Table S2). All but one of the six coarse sanidine crystals analyses yielded ^40^Ar/^39^Ar ages concordant with the ZDD age, with the youngest sanidine ^40^Ar/^39^Ar age of 7.2 ± 1.3 ka obtained in our own experiments overlapping with our ZDD eruption age within uncertainty. The ca. 7–20 ka range of sanidine ^40^Ar/^39^Ar ages^14,17^ is within the range of U–Th zircon crystallization age range of ca. 7.7–59 ka that necessarily predate the eruption.

Our ZDD eruption age is discordant with the ^14^C ages for the YC-162 and SG14-1058 tephra at the 2σ level, but concordant with the 7370 ± 30 cal yr BP age from the burnt branch at the base of the QXZ lava flow^3^. However, the ZDD eruption age is significantly older than the group of younger ages ranging from 4.3 ± 0.4 to 3.53 ± 0.3 ka obtained using Electron Spin Resonance, Uranium-series disequilibrium, and Thermoluminescences methods^15,19,20^.

The concordance of our new 7.0 ± 0.9 ka ZDD eruption age with the 7370 ± 30 cal yr BP from the burnt branch at the base of the QXZ lava and our sanidine ^40^Ar/^39^Ar ages connotes that the true eruption age of the QXZ is indeed < 8 ka, and we thus revise the age of the QXZ to 7.0 ± 0.9 ka.

## Discussion

Our new direct age for the QXZ has obvious implications for dating of the volcanic chrono-stratigraphy of a prominent and active volcano with a hazardous history, Holocene stratigraphic correlations in Asia/Japan, the productivity regarding magma accumulation and eruptive recurrence, and ultimately hazard assessments for the Changbaishan-Tianchi volcano.

An important issue that is revealed by this work is the discordance between the majority of the ^40^Ar/^39^Ar ages and the ZDD result. Only two sets of ^40^Ar/^39^Ar SCIH experiments on sanidine have yielded concordant results with our ZDD experiments, that of Heizler et al.^17^ and our own. Discordance between ZDD and ^40^Ar/^39^Ar ages is being increasingly recognized as several recent works have shown that ZDD consistently provides younger and more robust ages than corresponding ^40^Ar/^39^Ar ages on sanidine from important Quaternary eruptions^22–24^. This discordance partly reflects the difference in closure temperatures of the two systems. ZDD on volcanic rocks yields cooling ages (< 150 °C)^8^ that correspond to their eruption, provided that these rocks were preserved without subsequent thermal disturbance after deposition. (U–Th)/He is analogous to ^40^Ar-based dating methods, but the closure temperature of He in zircon (150–220 °C)^25,26^ is lower than that of Ar in commonly used K-bearing phases (e.g., biotite, sanidine, hornblende; 290–510 °C)^27^ for the same cooling rate. Hence, full retention of He in zircon starts at the time of eruption, although there are examples of crustal xenoliths in basaltic rocks where brief heating durations combined with the high amount of accumulated ^4^He in xenocrysts and/or in inclusions lead to (U–Th)/He dates that predate the eruption (ref.^28^). Similar issues may also cause ^40^Ar/^39^Ar ages in felsic volcanic rocks to be significantly older than the true eruption age, either as the entire population or more commonly as a spectrum of ages that are over dispersed relative to analytical uncertainties. The causes of this remain poorly understood, with excess Ar trapped within the crystals (ref.^29,30^) or melt inclusions (ref.^31–33^), and/or presence of antecrysts or xenocrysts that have not been fully degassed being commonly invoked (ref.^23,34–36^). In this respect the demonstrably older sanidine crystals in our ^40^Ar/^39^Ar experiments are particularly interesting and should be a target for future investigation, but will require deconvolving the possibility of excess ^40^Ar in melt inclusions and xenocrysts or phenocrysts^37^. Regardless of the potential causes, the two youngest ^40^Ar/^39^Ar results reveal that in some instances sanidine may have serendipitously by-passed the issues raised above, and that ^40^Ar/^39^Ar sanidine ages approach the data determined by ZDD. In the case of the QXZ eruption, the accuracy of the (U–Th)/He age is further supported by young zircon crystallization ages from U–Th disequilibrium method and concordance with robust ^14^C ages^16^. Danišík et al.^8^ expound on the potential advantages of the ZDD approach over not just the K–Ar and ^40^Ar/^39^Ar techniques, but also the commonly used radiocarbon, fission-track and luminescence methods.

An obvious outcome of this new age is that the QXZ does not straddle a 17 kyr Hilina-Pali/Tianchi magnetic reversal^1^ but may instead implicate a younger Holocene geomagnetic field excursion recorded in the QXZ comendite. Zhu et al.^4^ reported that the top and basal parts (their sample sites 1, 2 and 5) of the QXZ lava are normally magnetized with paleointensities of between 43 and 63 µT, but the central part of the flow, their sample site 3, are transitionally magnetized and sample site 4 is fully reversed with much lower paleointensities of 23.5 and 26.3 µT. The latter two samples have Virtual Axial Dipole Moments (VADM) of 3.4 × 10^22^ and 6.1 × 10^22^ A m^2^ respectively^1,4^. Although originally erroneously correlated with an excursion at 123 ka^4^, Singer et al.^1^ corrected this correlation to 17 ka based on their ^40^Ar/^39^Ar ages and posited that the low paleointensity along with reversed to transitional directions correspond to the Hilina Pali excursion previously dated at 19.3 ± 1.6 ka (^14^C calibrated)^38^. As their age for the QXZ matched this excursion, a new global magnetic field excursion named Hilina Pali/Tianchi was proposed. Our new age constraints now require that the classification of these anomalous magnetic directions and low intensity be further revised. Many works have attempted to pinpoint Holocene geomagnetic field variations (ref.^39–42^). These works provide a concise record of Holocene geomagnetic field variability, and they document that the lowest geomagnetic intensity in the Holocene occurred at around 7 ka, consistent with the QXZ record of a drop in intensity, where VADM was ~ 20% lower than today^1,4,39–42^. This low intensity was very similar to that for the mid Holocene record of the Levant (Israel, Syria, Jordan) and surrounding regions of SE Europe, the Caucasus, and N.Africa/Egypt^43^. However, there is no equivalent evidence for anomalous directions that could be considered excursional as recorded in the QXZ eruption studied by Zhu et al.^4^. Given that the QXZ excursion is interpreted to have occurred relatively rapidly, on the order of years^1^, one possibility is that this excursion was only locally recorded at Changbaishan-Tianchi, and not elsewhere, and it may have been too brief to be resolved by sedimentary records. Moreover, there is low probability to capture such a brief event in sparse contemporaneous volcanic and archaeomagnetic data set^44^. Hence, the current data do not categorically obviate a regional or even global excursion around ca. 7–8 ka if its duration was exceedingly short, and it is also possible that the data density and resolution of other studies of Holocene records have thus far overlooked any rapid paleomagnetic changes as recorded during the QXZ eruption. Either way, our new data warrant a more detailed examination of the Holocene record around 7–8 ka.

Correlations of the YC-162 tephra^2^ and SG14-1058 tephra^5^ recorded in Yuanchi lake and Lake Suigetsu have been used to promote the QXZ as another important CBS-TC-sourced marker horizon from central Japan to Northeast China (Fig. 1). Our new age, although just discordant at the 2σ level, is close enough to support this correlation, particularly when combined with the geochemical evidence. Geochemical similarities between proximal and distal glass compositions connote an eruption of some significance in this region^2,3,5^ although the magnitude of the eruption remains a matter of speculation. However, a preliminary estimate can be made on the basis of the known locations of the QXZ ash (Fig. 1). We make the conservative estimate that ash covered an elliptical area with semi major and minor axes of 900 km and 100 km respectively from Changbaishan-Tianchi, Yuanchi Lake, to Lake Suigetsu. Assuming a very conservative 1 cm thick deposit throughout this area, a volume of ~ 1 km^3^ can be estimated using the single isopach method of Legros^44^. The 1 cm average thickness is quite reasonable given that the thickness of the proximal tephra units amounts to well over 10’s of meters^12^, the “patchy tephra” in Unit 3 of the Yuanchi Lake core 30 km away is ~ 4 cm thick^2^ (1.62–1.58 m core depth), and the SG14-1058 cryptotephra 900 km from source is described as a “primary tephra isochron" and is found throughout a 1 cm thickness with a concentration of 5000 shards/gram (position F11 in the SG14 core; 28.6–29.4 cm^5^). Thus, although simplistic and based on limited available data, this minimum volume connotes that the explosive phase of the eruption was at least a 5 on the Volcanic Explosivity Index^45^. Sun et al.^2^ argue convincingly that the high concentration of QXZ glass recorded in Lake Suigetsu (> 5000 shards per gram of sediment) strongly suggests that the QXZ may be dispersed in a wider area than the very limited distribution currently known (Fig. 1)^2,5^. We follow Sun et al.^2^ and McLean et al.^5^ in asserting that this tephra layer potentially offers an important early Holocene marker horizon around East Asia and may help constrain the extent of important paleoenvironmental events like the “8.2 ka event”.

Attributing the timing of the QXZ eruption to regional deglaciation^1^ also has to be reevaluated in the light of our new age constraints. As the last glacial maximum in the northern hemisphere ended abruptly at 19–20 ka, the associated deglaciation cannot be the trigger of the QXZ and younger eruptions. It is possible that local glacial unloading may have occurred in the Holocene and triggered eruptions at the Changbaishan-Tianchi, but as far as we are aware there is no evidence to support this. Alternatively, the explosive eruptive tempo was driven by magma dynamics and magmatic evolution^3,10,16,37^. Three distinct eruptive episodes in the last 51 kyrs at the CBS-TC have been identified: the Tianwenfeng (Yellow Pumice; B-J) eruption at ca. 51 ka, the QXZ at ca. 7–8 ka (this work), and the ME at 946–947 CE^3^. These eruptive records have been linked to magmatic evolution from dominantly basaltic to trachyte to bimodal comendite-trachyte over the last 100 kyr, with trachyte-comendite interactions (recharge, mixing, and hybridization) playing a key role in the explosive eruption cyclicity^46^.

Our new data provide a zircon crystallization history perspective to the magma dynamics at the CBS-TC. ^238^U–^230^Th data reveal largely uniform zircon crystallization ages with an average of 18.1 ± 2.7 ka, omitting two older analyses with ca. 59 ka ages. This age is similar, albeit slightly older than the average zircon crystallization age of 12.2 ± 1.1 ka for the QXZ^16^, and indicates at least a 5–10 ka gap between zircon crystallization and eruption, a minimum period for the tempo of magma evolution given the fact that zircon only crystallizes in highly evolved comenditic magma compositions. Ra/Th isotopes suggest that the magma residence for the ME event is about 6–10 kyr^37^. This time scale is in close agreement with zircon residence timescales reported here and previously for the QXZ and may indicate a characteristic time scale for pre-eruptive magma residence at Changbaishan-Tianchi. The minor, but significant difference in rim crystallization ages found for our sample QXZD and that of Zou et al.^16^ could be due to preferential selection of large crystals for our ZDD study that may represent an earlier growth stage. The same magma containing ca. 12 ka zircon crystals was tapped during an explosive eruption at ca. 1 ka, but different zircon populations—younger and older than ca. 12–18 ka—are contained in ME deposits^16^. This implies that the QXZ comendite magma reservoir on the one hand contained even older evolved portions containing zircon antecrysts that may have only become remobilized prior to a very large eruption, and on the other hand, that the magma system was capable for rejuvenation and renewed zircon growth, possibly triggered by repeated recharge of magma into the pre-eruptive reservoir.

## Concluding remarks

New direct age ZDD and ^40^Ar/^39^Ar dating experiments, supported by a previous indirect ^14^C experiment now indicate an eruption age of 7.0 ± 0.9 ka for the QXZ of the CBS-TC. The revised age supports correlations with distal ash in Eastern China and Central Japan and establishes a significant (estimated at Volcanic Explosivity Index 5+) eruption that may provide a useful Holocene stratigraphic marker in East Asia. The new age indicates that the QXZ lava does not record a ca. 17 ka Hilina Pali/Tianchi geomagnetic field excursion but rather a heretofore unrecognized, maybe local, younger Holocene excursion at ca. 7–8 ka. Moreover, eruption triggering due to deglaciation after the last glacial maximum must be reevaluated. Comparison between U–Th zircon crystallization and the new eruption age indicates a protracted period of accumulation of the QXZ magma from ca. 18 ka to the eruption age, indicating remobilization and mingling of early formed crystals (antecrysts) and with autocrysts. Based on these results, pre-eruptive magma residence of ca. 7 ka for the Changbaishan-Tianchi magma system is found over the last two major eruption cycles.

## Methods

### ZDD experiments

A single sample (QXZD) from the distal flow-front (Fig. 1) of the proximal QXZ comendite clastogenic lava was processed for zircon by crushing, panning, magnetic separation, and picking under a binocular microscope. Most zircons were between 100 and 200 µm in length and have an aspect ratio of 1:3 to 1:4.

Zircon crystals were double-dated using combined U–Th disequilibrium and (U–Th)/He methods in the HIP Laboratory at Heidelberg University (Germany) and in the Western Australia ThermoChronology (WATCH) Facility at John de Laeter Centre (Curtin University, Perth, Australia), respectively, following the procedures detailed in Friedrichs et al.^47^ and Danišík et al.^22,48^.

Secondary ionization mass spectrometry (SIMS) analyses at the HIP Laboratory used a CAMECA ims 1280-HR ion microprobe tuned to sputtering positive secondary ions with a ~ 50 nA mass-filtered ^16^O^-^ beam focused to a ~ 30–40 µm diameter spot. Secondary ions were collected in dynamic multi-collection using Faraday cups for ^232^ThO^+^ and ^238^UO^+^, and electron multipliers for all other species (different Zr_2_O_3_^+^ species, ^230^Th^+^, and backgrounds). Relative sensitivity factors for ThO and UO were independently calibrated using measured ^232^Th and ^238^U, and accuracy was monitored by analyzing secular equilibrium zircon reference AS3 in replicate, for which an average (^230^Th)/(^238^U) = 1.011 ± 0.009 (mean square of weighted deviates MSWD = 0.75, n = 14) was obtained. Model ages for QXZD zircon rims were calculated as two-point isochrons anchored at the Changbaishan whole-rock composition from Zou et al.^16^ (^238^U)/(^232^Th) = 0.634 ± 0.010 and (^230^Th)/(^232^Th) = 0.711 ± 0.010.

For (U–Th)/He analyses at the WATCH Facility, zircon crystals were plucked out from the In mounts previously used for SIMS analysis, photographed and measured for dimensions in order to calculate alpha-ejection correction factor^49,50^, and individually transferred into niobium microtubes. Radiogenic ^4^He was extracted in an Alphachron instrument at ~ 1250 °C under ultra-high vacuum using a diode laser and its volume was measured by isotope dilution on a QMG 220 M1 Pfeiffer Prisma Plus mass spectrometer. A ‘re-extract’ was run after each sample to verify complete outgassing of the crystals. He gasses results were blank corrected by heating empty Nb tubes using the same procedure. After the ^4^He measurements, Nb microtubes containing the crystals were retrieved from the Alphachron, spiked with ^235^U and ^230^Th, and dissolved in Parr acid digestions vessels in two cycles of HF, HNO_3_ (cycle 1), and HCl acids (cycle 2) following the procedures described in Evans et al.^51^. Sample, blank, and spiked standard solutions were then diluted by Milli-Q water and analyzed by isotope dilution for ^238^U and ^232^Th, and by external calibration for ^147^Sm on an Element XR™ High Resolution ICP-MS. Total analytical uncertainty of uncorrected (U–Th)/He dates was calculated by propagating uncertainties of U, Th, Sm and He measurements. The uncorrected (U–Th)/He dates were Ft-corrected after Farley et al.^52^ assuming a homogeneous distribution of U and Th. The accuracy of zircon (U–Th)/He dating procedure was monitored by replicate analyses of Fish Canyon Tuff zircon (n = 4) measured as internal standard, yielding mean (U–Th)/He age of 28.5 ± 1.5 Myr ago (2σ), consistent with the reference (U–Th)/He age of 28.3 ± 1.3 Myr ago^52^. The Ft-corrected (U–Th)/He dates were then corrected for disequilibrium and pre-eruptive crystal residence by using the MCHeCalc software^53^ that requires as input parameters the Ft-corrected zircon (U–Th)/He ages and uncertainties, the zircon crystallization ages and uncertainties, and D_230_ and D_231_ parameters describing zircon-melt fractionation of Th and Pa relative to U. The D_230_ was calculated by dividing measured Th/U ratios of zircons by measured whole-rock Th/U. For D_231_ a value of 3.3 was adopted based on an average of published Pa/U zircon-rhyolite melt partition coefficient values^54–56^. Disequilibrium corrected (U–Th)/He dates (14 replicates per sample) were then used to calculate error-weighted mean and 95% confidence interval, which are interpreted as the representative eruption age (termed ZDD eruption age) and its uncertainty, respectively. Results for the U–Th disequilibrium and ZDD experiments are given in Table 2, Fig. 2, and Table S1.

### ^40^Ar/^39^Ar experiments

Hand samples for selected volcanic rock (Fig. 1; QXZC) were collected for ^40^Ar/^39^Ar dating. Mineral separates of sanidine and anorthoclase were produced at Oregon State University using conventional techniques including crushing, sieving (> 850 μm), washing, ultrasonic bathing and the use of magnetic separations using a FrantzTM model LB-1 magnetic separator. Mineral separates were cleaned by rinsing each sample with cold water, then washing in an ultrasonic cleaner for 15 min using triple distilled water (Milli-Q Water) then dried in a drying oven at 55 °C. Special care was taken to remove any alteration material from the groundmass using an intensive acid leaching procedure using a combination of HCl and HNO_3_ at different acid strengths and dried in a drying oven at 55°C^57^. The sanidine and anorthoclase concentrates were further treated with a 15% solution of HF for 7 min to remove adhering glass. The mineral concentrates were put through a solution of Lithium Heteropolytungstate (LST) using a density of 2.582 to float the anorthoclase and sink any possible plagioclase or anorthoclase with heavy inclusions. Samples were then washed with Milli-Q water and tried at 55 °C. Once the samples were dried, they were re-sieved between 250 μm to remove finer fractions produced from the ultrasonic cleaning. Final separates were obtained using a binocular microscope to obtain purities of > 99.9%.

Age determinations for sanidine and anorthoclase separates were obtained at the Oregon State University Argon Laboratory in Corvallis, Oregon, using incremental CO_2_ laser heating and/or Total Fusion Methods and analyzed on a multi-collector noble gas mass spectrometer. Anorthoclase separates, as well as sanidine flux monitors (FCT-2-NM with a calibrated age of 28.201 ± 0.023 Ma, 1σ; after Kuiper et al.^58^), were placed in irradiation package 16-OSU-02 and irradiated for 0.5 Megawatt hours in the CLICIT position at the TRIGA nuclear reactor at Oregon State University (OSU). Irradiated samples were loaded into Cu-planchettes in an ultra-high vacuum sample chamber and incrementally heated by scanning a defocused 25W Synrad CO_2_ laser beam at increasing laser powers in pre-set patterns across the sample, in order to evenly release the argon from the samples. Samples were analyzed using the total fusion method, where each crystal (set of 30 crystals for each sample) were heated at full power (26% power), or by the single crystal incremental heating method (SCIH). It must be noted that our laser system has not been calibrated for knowing exact temperatures. This system has not been calibrated for obtaining exact temperatures. After each heating step or total fusion run, and prior to analysis, reactive gases were cleaned for 90 s using a set of AP10 Zr-Al getters; 2 hot getters operated at 450 °C and 2 at room temperature (21 °C). Argon isotopic measurements were performed using a Thermo ScientificTM multi-collector ARGUS-VI noble gas mass spectrometer (spectrometer “D” at the OSU lab) that has 5 F collectors (fitted with a 10^12^ Ohm resistors for measurement of masses ^41^Ar and ^40^Ar, and with 10^13^ Ohm resistors for masses ^39^Ar, ^38^Ar, and ^37^Ar) and 1 ion-counting Cu-Be electron multiplier. This configuration allows to simultaneously measure all argon isotopes, with mass 36 on the multiplier and masses 37 through 40 on the four adjacent faradays. This configuration also provides the advantage of running in a full multi-collector mode while measuring the lowest peak (on mass 36) on the highly sensitive electron multiplier (CDD) located in a position next to the lowest mass faraday collector which, in turn, has an extremely low dark-noise and a very high peak/noise ratio.

All ages were calculated using the corrected value for the original Steiger & Jäger’s^59^ constant for total ^40^ K decay to ^40^Ar with a new value of 5.530 ± 0.097 × 10^–10^/yr (2σ) as reported by Min et al.^60^. For all other constants used in the age calculations we refer to Table 2 in Koppers et al^57^. Individual J-values for each sample were calculated by parabolic extrapolation of the measured flux gradient against irradiation height and typically give 0.06–0.13% uncertainties (1σ). Calculated ages used the assumed trapped ^40^Ar/^36^Ar ratio of 295.5. Incremental heating plateau ages and isochron ages were calculated as either plateau, mini-plateau or weighted mean with 1/σ2 as weighting factor^61^ and as YORK2 least-square fits with correlated errors^62^ using the ArArCALC v2.6.2 software from Koppers^63^ available from the following website http://earthref.org/ArArCALC/. All age uncertainties presented are 2-sigma. Sanidine total fusion ages (if obtained) are weighted mean probability ages also known as an Ideogram Plot. Ages are calculated without the uncertainty in J-value. Results are presented in Table S2 and shown in age spectrum are found in Fig. S2.

## Supplementary Information

Supplementary Figure S1.

Supplementary Figure S2.

Supplementary Table S1.

Supplementary Table S2.

## Data Availability

All data obtained in this study are available in the supplementary files of this manuscript.
