# Supplementary figures and images for: The Qixiangzhan eruption, Changbaishan-Tianchi volcano, China/DPRK: new age constraints and their implications
Source: Sci Rep. 2022 Dec 28;12:22485. doi: 10.1038/s41598-022-27038-5 (PMC9797483; doi:10.1038/s41598-022-27038-5)

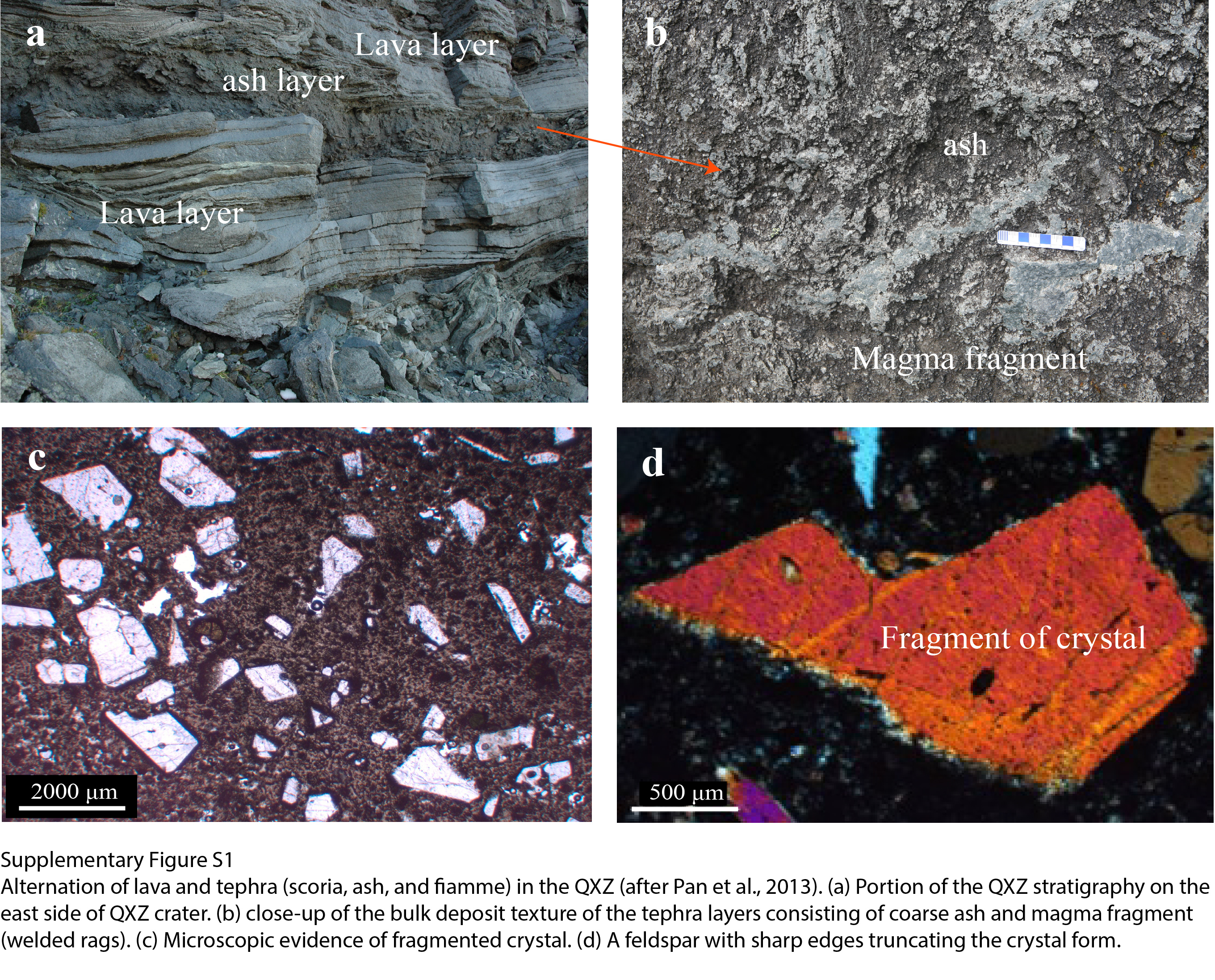

Supplement: Supplementary file 1 — Supplementary Figure S1. [file 41598_2022_27038_MOESM1_ESM.jpg]

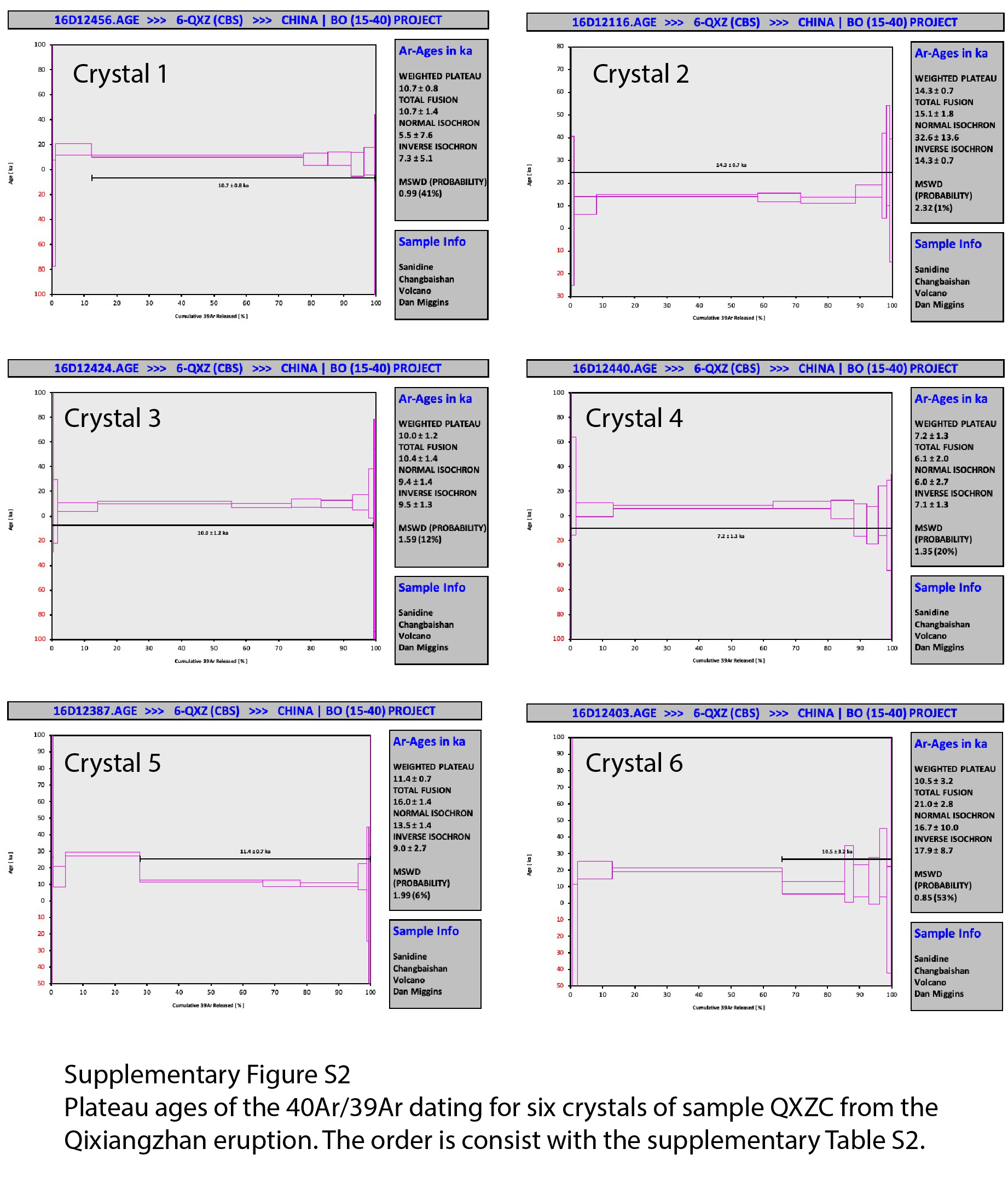

Supplement: Supplementary file 2 — Supplementary Figure S2. [file 41598_2022_27038_MOESM2_ESM.jpg]
